# Use of electronic cigarettes in the perioperative period: A mixed-method study exploring perceptions of cardiothoracic patients in Australia

**DOI:** 10.18332/tid/98957

**Published:** 2018-11-12

**Authors:** Nia A. Luxton, Patti Shih, Muhammad Aziz Rahman, Roger Adams, Ross MacKenzie

**Affiliations:** 1 Department of Psychology, Macquarie University, Sydney, Australia; 2 Australian Institute of Health Innovation, Macquarie University, Sydney, Australia; 3 Austin Clinical School of Nursing, La Trobe University, Melbourne, Australia; 4 Discipline of Physiotherapy, University of Sydney, Sydney, Australia

**Keywords:** e-cigarettes, coronary artery disease, lung cancer, preoperative, patient perceptions

## Abstract

**INTRODUCTION:**

Electronic cigarettes (e-cigarettes) may reduce tobacco use and achieve tobacco abstinence in the perioperative period of cardiothoracic surgery for patients who smoke. However, research on patients’ views on the role of e-cigarettes as a smoking cessation tool is lacking. This mixed-methods study explored perceptions on the use of e-cigarettes among current smokers and ex-smokers awaiting cardiothoracic surgery in Australia

**METHODS:**

A cross-sectional study and semi-structured interviews were conducted with 62 patients who were diagnosed with coronary artery disease or lung cancer and were scheduled for elective cardiothoracic surgery at six metropolitan hospitals in Sydney. Data were collected on demographic characteristics, smoking history, surgical risk index, self-efficacy, interest in, perceived benefits of, and barriers to using e-cigarettes in the perioperative period

**RESULTS:**

Current smokers reported significantly higher interest in the use of e-cigarettes (p=0.008), and perceived fewer barriers (p=0.048) and more health benefits (p=0.079), compared to ex-smokers. Current smokers considered e-cigarettes to be either a safer alternative to tobacco or a novel method for quitting. Recent ex-smokers, defined as those who quit 2–8 weeks, were a distinct group with high nicotine dependency, a long history of smoking, and multiple failed quit attempts. Compared to longer-term ex-smokers (8–52 weeks quit), recent ex-smokers were more interested in e-cigarettes (p=0.029) and considered e-cigarettes a useful aid to prevent relapse in the lead up to surgery and to manage their nicotine cravings.

**CONCLUSIONS:**

E-cigarettes may be considered a short-term novel aid and a bridge to evidence-based methods to reduce harm from continued tobacco use for some patients awaiting cardiothoracic surgery for coronary artery disease or lung cancer. This study presents reasons why patients awaiting cardiothoracic surgery may enquire about or use e-cigarettes, which will help clinicians identify those who need more consistent, sustained cessation support.

## INTRODUCTION

Electronic cigarettes (e-cigarettes) may potentially offer a safer alternative to tobacco smoking^1,2^ and assist in cessation^3,4^. Methods for smoking cessation are particularly crucial for patients diagnosed with lung cancer or coronary artery disease (CAD) who require cardiothoracic surgery, as continued tobacco smoking increases the risk of postoperative complications, disease recurrence and death^5-7^. Studies in the US involving surgical patients in the perioperative period suggest that e-cigarettes are a feasible and acceptable way to reduce tobacco use in the perioperative period^8,9^. However, the complex Australian regulations on e-cigarettes, and opposition by government and health authorities, have limited both the use of e-cigarettes and research studies examining e-cigarettes as a smoking cessation tool in the clinical setting^10^.

International research shows that inadequate access to cessation support can contribute to hospitalised patients’ interest in using e-cigarettes to help quit smoking^11^. In view of the need to reduce the harm caused by tobacco use in patients with cancer or CAD, and the recurrence of primary disease and development of secondary disease, studies have shown that e-cigarettes can address a number of behavioural and psychosocial factors contributing to relapse^12^ and are being used to reduce or quit smoking^13-15^, reduce the harm of tobacco use^16^, and reduce nicotine cravings^17^. In Australia, smoking rates are low, yet smoking prevalence and the incidence of lung cancer and CAD is higher among older, disadvantaged, or lower socioeconomic groups^18-20^. For some of these patients with lung cancer or CAD, e-cigarettes may have a role to help reduce or quit smoking.

The perioperative period is a ‘teachable moment’ that leads to permanent smoking cessation for many patients^21,22^. However, patients with lung cancer or CAD may continue to smoke due to stress, lower readiness or motivation to quit, lower self-confidence in being able to quit, prior failures to quit using other evidence-based cessation methods, or the lack of smoking cessation advice or support^23,24^. Recent surveys in Australia have shown that current smokers report higher levels of awareness, interest in and use of e-cigarettes as aids to quit smoking compared to ex-smokers^25,26^. Also, a study of hospitalised smokers in Australia found that while few of the 600 participants reported using e-cigarettes in their previous quit attempts, almost one-third showed interest in using e-cigarettes in any future attempts^27^. However, no studies have specifically focused on patients in cardiothoracic surgery.

This mixed-methods study aims to assess the perceptions of patients with lung cancer and CAD on the use of e-cigarettes to reduce tobacco smoking in the perioperative period of cardiothoracic surgery.

## METHODS

### Design

A convergent mixed-methods study, using a survey and a semi-structured interview, was conducted between October 2015 and November 2016, to assess e-cigarette perceptions in the cardiothoracic surgery perioperative period. Face-to-face interviews added depth to the survey responses and explored patients’ views on e-cigarettes to corroborate and interpret the findings^28^.

Ethics approval was obtained from the Northern Sydney Local Health District Hospital Ethics Committee (LNR/15/HAWKE/356), and the six Sydney hospitals. The three public tertiary referral hospitals and three private hospitals in the study were responsible for 43% of cardiothoracic surgery cases in NSW in 2016, with patients from urban, rural and remote areas, thereby increasing generalisability^29^.

### Study population

#### Inclusion and exclusion criteria

The study population comprised patients aged 18 years or older, who had a self-reported smoking status of either every day, most days, or were recent ex-smokers of < 12 months at time of attendance at either a preadmission clinic (PAC) or inpatient ward, at one of the six hospitals in Sydney. All patients had a diagnosis of lung cancer or CAD and were scheduled for elective coronary artery bypass surgery (CABG) or thoracic surgery related to their cancer. Patients with a current or former history of smoking for one year, or less, at time of preoperative interview were purposefully included, as under-reporting of smoking status is common due to fear of medical judgement^30,31^ and surgery cancellation^32^ and the period from diagnosis to elective cardiothoracic surgery in NSW can vary from days to months^33^.

Eligible patients were identified by clinical personnel, either from a patient’s hospital records or from discussions with the patient at the preadmission clinic, or in a cardiothoracic ward. Each patient that was included in the study gave written informed consent. After the survey and interview, each patient was offered participant information and advice in accordance with NSW Health smoking cessation guidelines^34^.

### Data collection

The patient survey (Appendix A) was modelled on two studies of non-cardiothoracic patients in preadmission clinics in the US^8,9^ with two additional questions and so comprised 39 items. The online survey was administered via a touchscreen tablet computer (iPad, Apple Inc), and recorded using Qualtrics software (Qualtrics, Provo, UT). Immediately after completion of the survey, a semi-structured interview, based on the same questions as the survey, was conducted to gain a deeper understanding of patients’ beliefs and perceptions about e-cigarettes, both in general and in the preoperative period of cardiothoracic surgery. Interviews were digitally recorded, and each interview continued until the patient had no new information to add. The mean completion time for the survey and interview in total was 45 minutes (range 27–60 minutes).

Data collection ceased after 62 patients, when ‘theoretical saturation’ was reached, the point at which no new concepts emerged from reviewing successive data from a sample that is diverse in pertinent characteristics and experiences^28^. All surveys and interviews were administered and conducted in a private area of a PAC or ward by the same researcher (NAL), a senior physiotherapist and academic with over 20 years’ international clinical experience in cardiothoracic surgical care. The researcher was independent of the other health professionals involved in patients’ perioperative care.

### Measures

#### Participant characteristics

Characteristics recorded included age, gender, ethnicity, education, residential area and baseline smoking history, as well as current smoking status, previous quit attempts and methods, and the Fagerström test for nicotine dependence (FTND)^35^.

Self-efficacy, referring to a patient’s perceived ability to stop smoking in the perioperative period, was assessed using questions about a patient’s intention to quit smoking in the future (yes/no) and the likelihood of abstinence after surgery, using a five-point Likert scale from one (very unlikely) to five (very likely) (Questions 18 and 19, Appendix A). Surgical health risk index (SHI) was used to assess the knowledge of health risks of tobacco smoking related to surgery, and the risks of tobacco smoking on perioperative complications. The four questions of the SHI (Appendix A) were scored by summing the number of ‘agree’ responses. These measures have been previously used in perioperative patient populations^8,9,36^. Patients’ perceptions of e-cigarettes were examined using previously developed questions^8,9^ (Appendix A) and included: four items to assess interest in using e-cigarettes to reduce perioperative cigarette use; four items to assess perceived benefits of perioperative e-cigarette use; and four items to assess perceived barriers to perioperative e-cigarette use. Interest referred to patients’ beliefs about, and willingness to try, e-cigarettes to help reduce or abstain from tobacco cigarettes around the time of surgery. Perceived benefits referred to whether e-cigarettes could help patients cope without tobacco cigarettes around the time of surgery as well as to do better before and/or after surgery. Perceived barriers referred to safety, cost, difficulty, and whether the patients had other concerns rather than try e-cigarettes around the time of surgery. Items and categories were assessed using a five-point Likert scale from strongly disagree to strongly agree.

### Data analysis

The surveys and interviews were initially analysed separately, and then the results compared with the qualitative findings helping to inform and better frame the quantitative survey findings^28^. Characteristics of patients, including smoking history, self-efficacy and SHI, interest in, perceived benefits of and perceived barriers to e-cigarette use are summarised in Table 1. Patients were categorised as current smokers (selfreported smoking occasionally or daily in the 2 weeks prior to research interview), recent ex-smokers (selfreported smoking occasionally or daily until cessation 2–8 weeks prior to interview) and longer-term ex-smokers (self-reported smoking occasionally or daily until cessation 8–52 weeks prior to interview). Patients with prior e-cigarette use were termed ever-users and those with no prior use were termed never-users.

**Table 1:** Characteristics of patients (N=62 )

*Characteristic*	*Class/statistic*	*Current smoker (n=32 )*	*Recent ex-smoker (n=18 )*	*Longer term ex-smoker (n=12 )*	*Total N=62*
Sex	Male	25 (78%)	9 (50%)	11 (92%)	46 (74%)
	Female	8 (35%)	7 (39%)	1 (8%)	16 (26%)
Age (y)	25–40	4 (12%)	0 (0%)	0 (0%)	4 (7%)
	40–54	8 (25%)	4 (22%)	0 (0%)	12 (19%)
	55–64	11 (34%)	2 (11%)	6 (50%)	21 (31%)
	65–84	9 (28%)	12 (67%)	6 (50%)	27 (43%)
Highest level of education	< Year 12	16 (42%)	6 (33%)	3 (25%)	25 (40%)
	Year 12	10 (31%)	11 (61%)	8 (67%)	29 (47%)
	Tertiary	6 (19%)	1 (6%)	1 (8%)	8 (13%)
Age started smoking	< 18 years	21 (66%)	10 (56%)	9 (75%)	40 (65%)
	≥ 18 years	11 (34%)	8 (44%)	3 (25%)	22 (35%)
Location	Metropolitan	17 (53%)	12 (67%)	9 (75)	38 (61%)
	Regional	15 (47%)	7 (38%)	9 (25)	24 (39%)
Ethnicity	Australian	6 (19%)	4 (22%)	2 (17)	12 (19%)
	ABTSI	5 (16%)	1 (5%)	0 (0%)	6 (10%)
	European	16 (50%)	9 (50%)	5 (42)	30 (48%)
	Other	5 (16%)	4 (22%)	5 (42%)	14 (22%)
Type of surgery	Thoracic	8 (25%)	4 (22%)	3 (25%)	14 (22%)
	Cardiac	24 (75%)	14 (78%)	9 (75%)	48 (77%)
Current number of cigarettes	<10/day	23 (72%)	0 (0%)	0 (0%)	23 (37%)
	≥10/day	10 (32%)	0 (0%)	0 (0%)	10 (16%)
Plan to stay off cigarettes after surgery	Yes	28 (90%)	17 (94%)	11 (92%)	56 (90%)
The likelihood of staying off tobacco smoking after surgery (self-efficacy)	Very likely	6 (18%)	10 (56%)	8 (67%)	24 (39%)
	Likely	12 (38%)	4 (22%)	3 (25%)	19 (30%)
	Undecided	8 (25%)	4 (22%)	1 (8%)	13 (21%)
	Unlikely	6 (19%)	0 (0%)	0 (0%)	6 (10%)
	Very unlikely	0 (0%)	0 (0%)	0 (0%)	0 (0%)
Prior quit attempt in last year	Yes	20 (46%)	15 (35%)	8 (19%)	43 (69%)
Prior use of e-cigarettes	Yes	6 (20%)	7 (41%)	2 (13%)	15 (24%)
Current use of e-cigarettes	Yes	0	1 (6%)	0	1 (1%)
Fagerström test for nicotine dependence^a^	Mean (SD)	4.1 (1.9)	4.9 (1.7)	3.0 (1.4)	4.1 (1.9)
Surgical health risk index (four items, max score = 4)^b^	Mean (SD)	2.8 (1.5)	3.2 (1.2)	3.1 (1.3)	2.9 (1.4)
Sum of interest in e-cigarettes around the time of surgery (four items, max score = 20)^b^	Mean (SD)	12.4 (4.1)	9.4 (4.0)	8.3 (2.8)	10.7 (4.2)
Perceived benefits of using e-cigarettes around the time of surgery (four items, max score = 20)^b^	Mean (SD)	12.5 (4.1)	9.6 (4.0)	9.5 (3.0)	11.1 (4.1)
Perceived barriers to using e-cigarettes around the time of surgery (four items, max score = 20)^b^	Mean (SD)	11.8 (2.0)	12.6 (2.0)	13.6 (1.5)	12.3 (2.0)

a Higher scores indicate more nicotine dependence.

b Descriptions and indices calculated as described in the methods. ABTSI: Aboriginal or Torres Strait Islander descent.

One-way analysis of variance (ANOVA) was used to examine in-group differences on surgical risk, interest, perceived benefits, perceived barriers and FTND according to smoking status (current/recent ex-smoker/longer-term ex-smoker, ever/never e-cigarette use). Self-efficacy in terms of intention to quit after surgery was also examined according to smoking status and perceptions of e-cigarettes using ANOVA. Chi-squared tests were used to examine association between e-cigarette perceptions and use with demographics and self-efficacy. All tests were performed using SPSS Version 21 (IBM Corporation), and p<0.05 was defined as statistically significant.

Analysis of the survey data revealed different perceptions of e-cigarettes in the perioperative period according to patients’ smoking status (Table 1) but not by disease or other demographic characteristics. These findings formed the predetermined coding structure for the qualitative interview data to identify key reasons and explanations of patients’ perceptions towards e-cigarette use. This approach to content analysis allowed the integration and connection of the quantitative and qualitative data in the study^37,38^. NVivo 11 (QSR International Pty Ltd, Melbourne, 2018) was used for interview data organisation and retrieval^39^. The following techniques were used for scientific rigour: audiotaping and independent preparation of the transcripts; standardised coding and data analysis; use of researchers with diverse clinical and statistical backgrounds; and the creation of an audit trail to document analytic decisions^40^.

## RESULTS

### Baseline characteristics of patients

Most patients were male, older than 65 years, and had started smoking under the age of 18 years (Table 1). Of the 62 patients, over half were current smokers, and one-third of all patients had quit tobacco smoking less than eight weeks ago (recent ex-smokers). Most patients had made at least one quit attempt previously, primarily through abrupt cessation, with over a third of patients making multiple attempts using other methods such as medical advice from their general practitioner, NRT (patches, gum or inhaler) and varenicline. Eleven patients had never previously made a quit attempt. Of the 15 patients who had prior experience with e-cigarettes, six had bought them to try to quit tobacco, and eight had been given them by family or friends. Almost all patients (94%) were recruited from public hospitals, as patients in the private hospitals self-reported quitting more than a year before their preadmission interview.

Patients were scheduled for cardiothoracic surgery within 4 weeks (±4 weeks) at the time of research interview. Over half reported being current smokers (1±0.2 days), one-third were recent ex-smokers (7±1.5 weeks), and a fifth were longer-term ex-smokers (34±10.5 weeks). Most patients (90%) intended to stay off smoking after surgery, however, current smokers had lower self-efficacy to abstaining from tobacco cigarettes after surgery, with fewer current smokers reporting that they were likely or very likely to remain abstinent after surgery (56%) compared to longer-term ex-smokers (83%) (p=0.021, OR=0.26, 95% CI: 0.078–0.843).

**Figure 1:**
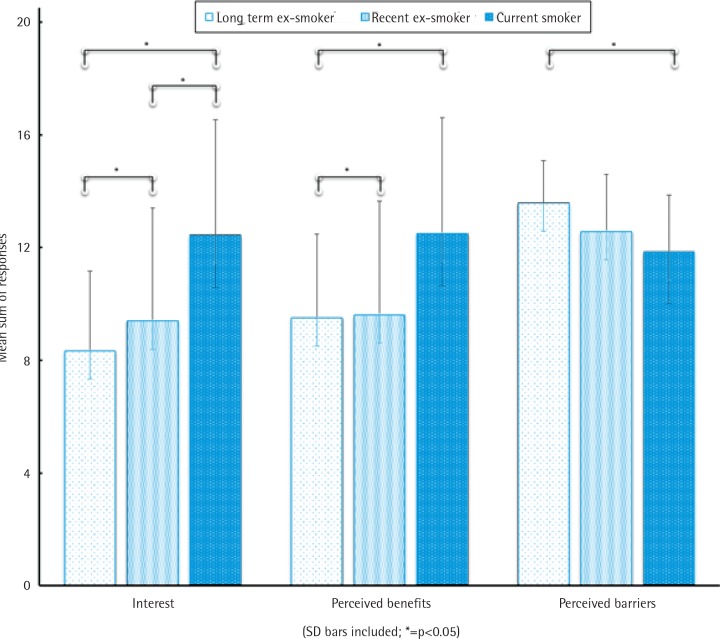
Interest in, perceived benefits of and barriers to the use of e-cigarettes in the perioperative period of cardiothoracic surgery

Interestingly, the perceived benefits of, barriers to and interest in e-cigarette use in the perioperative period of surgery significantly differed according to a patient’s smoking status (Figure 1) and prior experience with e-cigarettes. Appendix B summarises the perceptions of and interest in e-cigarettes during the perioperative period by smoking status, in a similar manner to the US studies on which this study was modelled^8,9^.

### Interest in e-cigarette use in the perioperative period

Both current smokers and recent ex-smokers had significantly higher levels of interest in the use of e-cigarettes around the time of surgery compared to longer-term ex-smokers (Figure 1). The interviews revealed that the interest in e-cigarettes by patients who were either smoking or had recently quit reflected their desire to stop smoking in the perioperative period. The primary reason for interest in e-cigarettes was their novelty compared to other cessation methods. Many patients had tried to quit using abrupt cessation, NRT in its various forms, or pharmacotherapy in previous years and following their recent diagnosis, surgeon-patient interview, or hospitalisation. Of the 32 current smokers, 12 (38%) had been unsuccessful with their previous chosen method, which included: varenicline (stopped due to reported adverse psychological effects); over-thecounter NRT patches without assistance; or abrupt cessation (the most common method previously used). Of the 18 recent ex-smokers, 12 (67%) were not confident in their ability to abstain using their current method, which was predominantly abrupt cessation and over-the-counter NRT patches without assistance. For eight of the 12 recent ex-smokers, this was their second or third attempt at quitting, but none reported seeking or being offered any formal support, such as from a Quitline telephone service or from a tobacco cessation counsellor.

Eleven (34%) of current smokers felt that the benefit of e-cigarettes was the potential to fill the void created by quitting tobacco cigarettes, replicate the hand-to-mouth action or continue the habits associated with smoking, as highlighted by a patient still smoking at the time of the PAC interview:

‘It’s the holding thing that e-cigarettes might help me with. That’s what I’ve missed with the other [NRT].

*When I was in hospital I was in acute [coronary] care – every ten minutes I was getting up and going out for a cigarette.’* (P.15)

Recent ex-smokers reported higher nicotine dependency (FTND=4.89±0.39, p=0.03) in the survey, compared to current smokers or longerterm ex-smokers. Of the 18 recent ex-smokers, eight (44%) expressed strong feelings of nicotine cravings during the interviews, and were interested in the role of e-cigarettes to reduce their risk of relapse back to tobacco smoking before surgery:

*‘I would try one if I knew it would help me with all my cravings. I was going to buy two packets [of tobacco] and put them in the cupboard, because no one will buy for me. I don’t know what to do. It’s always been my best friend.’* (P.23)

There were patients who were uninterested in e-cigarettes. All longer-term ex-smokers professed to having neither the knowledge nor the interest in e-cigarettes as they had quit tobacco smoking and did not want to create another habit or nicotine dependence:

*‘I wouldn’t try e-cigarettes again. I wouldn’t go back to using any cigarettes because I don’t want the nicotine addiction again.’* (P.27)

Other reasons for their lack of interest in e-cigarettes included: e-cigarettes were an ineffective method of quitting, based on personal experiences (2 recent ex-smokers and 2 current smokers); the patients were more confident in their ability to remain off tobacco without any cessation aid due to the support of family (2 recent ex-smokers); the patients were not interested in quitting smoking before or after surgery (4 current smokers). These patients had CAD and either had family members who had continued smoking after cardiac surgery in previous years, concurrent illicit drug use, or comorbidities such as HIV and depression.

### Perceived benefits to using e-cigarettes in the perioperative period

Current smokers and recent ex-smokers perceived significantly more benefits in the use of e-cigarettes around the time of surgery compared to longer-term ex-smokers (Figure 1). There was a general uncertainty about the contents of e-cigarettes, yet a higher proportion of current smokers (50%) and recent ex-smokers (48%) considered e-cigarettes to be better for their health compared to tobacco smoking in the perioperative period, which they knew would cause ‘problems’ during or after surgery. Again, recent ex-smokers considered the benefits of e-cigarettes were a means of tobacco harm reduction and relapse prevention:

*‘I don’t know if it will help me do better after surgery, but I would try one as I must try to stay off cigarettes. I don’t know how long I can stay off smoking.’* (P.42)

However, current smokers’ views were more pragmatic, with e-cigarettes deemed a healthier form of nicotine delivery compared to smoking:

*‘I have been tempted to try one. You’re not getting rid of the nicotine but you’re getting rid of all the other crap which is doing you more damage than the nicotine.’* (P.25)

Patients who were neutral to or disagreed with the survey questions on e-cigarette benefits (Appendix A) had diverse reasons. Among the 32 current smokers, 6 (19%) reported that they enjoyed tobacco smoking, knew ‘their enemy’ in tobacco and preferred it to any alternative, whereas among the 18 recent ex-smokers, 6 (33%) were wary about e-cigarettes and their ability to create a personal temptation to relapse, particularly among those missing the taste or act of smoking:

*‘It might lead me back to smoking. It’s the same trigger isn’t it? It’s the same – you’re inhaling, you’re holding something.’* (P.9)

Negative views of the benefits of e-cigarettes were expressed by 4 of the 15 patients with prior use of e-cigarettes due to either personal adverse symptoms of nausea or coughing at first use, or negativity from family or the media:

‘It made me very healthy for a year. But when they said to me that it [continuing vaping] was very dangerous, I gave it up straight away. We heard it on the news. That’s why I stop it and went back to smoking.’ (P.32)

### Perceived barriers to using e-cigarettes in the perioperative period

Longer-term and recent ex-smokers perceived more barriers to the use of e-cigarettes than current smokers (Figure 1), with safety and the unknown risks of e-cigarettes and their constituents commonly cited as a barrier. This view was more prominent among those patients with prior negative experiences of e-cigarettes, recent ex-smokers who viewed e-cigarettes as a temptation back to smoking (55%), and among 83% of the 12 longer-term ex-smokers who had reported little knowledge or interest in e-cigarettes:

*‘I don’t know about them what I can say, it’s smoking whether it’s electronic or not, it is dangerous. If you’re smoking that’s not good for your health.’* (P.30)

Other longer-term ex-smokers (7%) regarded e-cigarettes a backwards step, implying that the person did not really want to quit:

*‘I think they’re disgusting, they’re smoking cigarettes anyway. What’s the point? Users have no willpower [to quit].’* (P.60)

The barrier of learning how to use an e-cigarette was expressed mostly by older patients (age 65–84 years) who had been smoking for over 20 years, were either current smokers (21%) or had recently quit (15%) and had no e-cigarette experience. However, among most other current smokers, fewer barriers to e-cigarette use around the time of surgery were perceived, with 25 (79%) strongly stating that e-cigarettes had to be safer than tobacco smoking:

*‘What aren’t you getting? 90% of the chemicals that a cigarette has.’* (P.11)

## DISCUSSION

This is the first Australian study to report on the perceptions of e-cigarettes of patients with lung cancer and CAD awaiting cardiothoracic surgery. Patients’ smoking status likely predicts their perceptions of benefits, barriers and interest in e-cigarettes as a smoking cessation tool in the perioperative period. Patients who were current smokers or had recently quit showed particular interest in e-cigarettes to improve their surgical outcomes and to reduce tobacco harm compared to longer-term ex-smokers. E-cigarettes were perceived negatively by ex-smokers who either firmly identified themselves as a ‘non-smoker’ or were more uncertain of the efficacy of e-cigarettes in helping people remain abstinent from smoking. For current smokers and recent ex-smokers who feel incapable of negotiating the constant challenge of perioperative tobacco abstinence and have previously failed a quit attempt or have relapsed in the perioperative period, completely switching to e-cigarettes, coupled with proactive cessation support and counselling, may be a novel method to achieve and maintain tobacco abstinence.

Findings from studies in the US^8,9^ have also indicated that current smokers, irrespective of their prior e-cigarette use, were more likely to be interested in e-cigarettes to reduce tobacco use prior to surgery. However, these studies did not include self-reported ex-smokers, or patients at different stages of preoperative quit attempts. This study has identified recent ex-smokers who quit 2–8 weeks prior to preoperative research interview as a *distinct group* that should be specifically considered by clinicians and policy-makers as candidates for e-cigarette assisted smoking cessation. Whilst these patients demonstrated motivation to quit smoking prior to surgery, they also displayed characteristics associated with a propensity for relapse, such as higher nicotine dependency, a long history of smoking, multiple failed quit attempts and low self-efficacy compared to longer-term ex-smokers. These recent ex-smokers had higher expectations that e-cigarettes would maintain their quit attempt by providing behavioural cues or offering a sufficient tobacco substitute. However, whilst some of these patients had used NRT patches in prior quit attempts, it had not been in conjunction with oral NRT, or with any means of formal cessation support. Therefore, in a post-quit period of approximately one month, patients who are demonstrating high nicotine dependence and who are at high risk of relapse^41,42^ could be offered either NRT or e-cigarettes, together with smoking cessation advice and support as a short-term aid to sustain their quit attempt, maintain tobacco abstinence and reduce surgical risk.

The positive attitudes of current smokers to e-cigarettes as a means to quit smoking in this study mirror recent surveys of representative samples of Australian national^43^ and State^25^ populations and studies amongst lower socioeconomic groups, including people with substance use disorders and mental illness in Australia^44^. There was uncertainty about the safety and risks of e-cigarettes amongst most patients in this study, which is partly attributed to the stringent regulations on the sale of nicotine-containing e-cigarettes in Australia^45^. However, amongst current smokers or recent ex-smokers, e-cigarettes were considered a means of tobacco harm reduction and a tool to reduce or quit smoking, particularly when other methods of cessation had not led to personal tobacco cessation. Prior unsuccessful quit attempts were also suggested as a reason for high levels of interest in e-cigarettes for future quit attempts among hospitalised smokers^27^. However, as e-cigarettes are not included in Australian clinical guidelines for smoking cessation^46^, e-cigarettes were not offered as a cessation method^47^, thus the effectiveness of e-cigarettes as a novel method for smoking reduction or cessation amongst the patient population has not been examined in Australia. Given that the incidence of tobacco-related diseases such as lung cancer and CAD are higher amongst older and disadvantaged populations in Australia, the positive perceptions of e-cigarettes found in this study and other studies^27,44^ indicate that switching to e-cigarettes, coupled with extended cessation support, may be a feasible, novel method^48^ to quit tobacco by people with comorbidities for one to three months after hospitalisation^24^.

No significant differences were found between a patient’s disease and their perceptions of e-cigarettes in this study. However, the characteristics and perceptions of patients awaiting cardiothoracic surgery are comparable to those of international studies of patients recently diagnosed with cardiothoracic diseases or in the perioperative period of non-cardiothoracic surgery. For example, in the US e-cigarette use was reported among post-acute coronary syndrome patients who had reported more lifetime quit attempts and lower confidence in their ability to quit^14^, and amongst patients with lung cancer and high nicotine dependence^13^. Also in the US surgical field, patients were interested in e-cigarettes to reduce tobacco consumption in the perioperative period, particularly if they had never used e-cigarettes^8^. Less interest was reported among patients who had unmet expectations of the devices to help them quit tobacco smoking^8,9^, or were not interested in quitting tobacco smoking.

While research into the awareness and use of e-cigarettes by the general population is increasing in Australia, this study adds to the limited literature investigating the use and perceptions of patients with tobacco-related comorbidities. Diagnoses of lung cancer or CAD, hospitalisation and surgery all serve as powerful ‘teachable moments’ for behavioural change^21^, but some patients continue to smoke or relapse for a variety of reasons. The findings of this study and our previous work^23^ illustrate that despite a patient’s motivation to quit prior to surgery, the delivery and use of evidence-based methods do not necessarily lead to cessation success. Therefore, e-cigarettes coupled with consistent, proactive cessation support both before and after surgery may engage more patients in a quit attempt, be a bridge to evidence-based cessation methods and lead to longer-term, permanent postoperative cessation.

### Limitations

The size of our sample, the sampling method and the complex regulatory environment in Australia limits the generalisability of the findings beyond Australia. Similarly, although the sample included patients from diverse backgrounds and demographics from the most populous city and State in Australia, it does represent a very small percentage of the cardiothoracic surgeries by the Australian population. However, using a mixed-methods approach allowed better understanding both of patients’ opinions about e-cigarettes and patients’ influences on achieving or maintaining tobacco abstinence in the preoperative environment. Fewer patients were recruited with lung cancer and from private hospitals. This was due to smaller number of patients self-reporting smoking in the private PACs, and fewer lung cancer patients attending cardiothoracic surgical PACs at five of the six hospitals. Nonetheless, a strength of this study is the realistic representation of patients who smoke in Australia, their honesty about their smoking and psychosocial histories, and the time given by patients in each interview, allowing a deep insight into their views on tobacco and e-cigarette use. Since the interviews were conducted however, there has been an Australian parliamentary inquiry^49^ into e-cigarettes, which resulted in more media discussion about their safety and efficacy as smoking cessation aids. Whilst no changes were made to current regulations on e-cigarette sale and use in Australia, the intense media discussions may have increased patients’ awareness of the existence and/ or altered some of the patients’ views about the risks and benefits of e-cigarettes.

## CONCLUSIONS

This study has found that patients with lung cancer and coronary artery disease, who either currently smoke or have recently quit, have positive perceptions of e-cigarettes for reducing tobacco harm in the perioperative period. Patients identified specific roles for e-cigarettes, predominantly as an alternative method when other cessation methods had failed, or as a tool to prevent relapse for those struggling to maintain preoperative tobacco abstinence. The study provides insights for clinicians involved in the care of cardiothoracic surgical patients on why patients may enquire about or use e-cigarettes. It will help clinicians enhance the teachable moment of surgery by offering proactive, long-term evidence-based perioperative cessation support to patients awaiting cardiothoracic surgery, irrespective of their current or recent smoking status.
