# Supplementary material for: Use of electronic cigarettes in the perioperative period: A mixed-method study exploring perceptions of cardiothoracic patients in Australia
Source: Tob Induc Dis. 2018 Nov 12;16:53. doi: 10.18332/tid/98957 (PMC6659508; doi:10.18332/tid/98957)
Supplement: Supplementary file 1 [file TID-16-53-s1.pdf]

Appendix A: Survey of patients (Qualtrics, iPad)

Q1 Where do you live?
- ❍ Metropolitan Sydney
- ❍ Central Coast and surrounds
- ❍ Rural NSW
- ❍ Remote NSW
- ❍ Other ____________________

Q2 What is your age?
- ❍ 18-24
- ❍ 25-44
- ❍ 40-54
- ❍ 55-64
- ❍ 65-84
- ❍ 85+

Q3 What is your gender?
- ❍ Male
- ❍ Female

Q4 What is the highest educational qualification you have obtained?
- ❍ Less than Year 12
- ❍ Year 12
- ❍ Certificate
- ❍ Diploma
- ❍ Bachelor's degree
- ❍ Postgraduate qualification
- ❍ Doctorate

Q5 What is your ethnicity?
- ❍ Aboriginal or Torres Strait Islander
- ❍ European
- ❍ Asian
- ❍ Middle East
- ❍ African
- ❍ Other ____________________

Q6 At what age did you start smoking?
- ❍ < 18 years
- ❍ 18-35 years
- ❍ 35 years and over

Q7 Do you currently smoke?
- ❍ Every day
- ❍ Some days
- ❍ Not at all

Q8 When did you smoke your last cigarette?
❍ Today
❍ Yesterday
❍ Less than 2 weeks ago
❍ Less than 4 weeks ago
❍ More than 4 weeks but less than 8 weeks ago
❍ Within the last year

Q9 The following 6 questions (questions 9-14) will ask about your current smoking habits:
How soon after waking do you smoke your first cigarette?
❍ Within 5 minutes
❍ 5-30 minutes
❍ 31-60 minutes

Q10 Do you find it difficult to refrain from smoking in places where it is forbidden? E.g. Church, Library, Hospital
❍ Yes
❍ No

Q11 Which cigarette would you hate to give up?
❍ The first in the morning
❍ Any other

Q12 How many cigarettes a day do you smoke?
❍ 10 or less
❍ 11-20
❍ 21-30
❍ 31 or more

Q13 Do you smoke most frequently in the morning?
❍ Yes
❍ No

Q14 Do you smoke even if you are sick in bed most of the day?
❍ Yes
❍ No

Q15 Have you tried to quit smoking in the last year?
❍ Yes
❍ No

Q16 If you have made a quit attempt, what is the longest time you have quit for?
❑ Never
❑ < 1 month
❑ 1-6 months
❑ >6 months
❑ Don't know

Q17 If you have made a quit attempt how did you do it? Tick all that apply.

- ❑ 'Cold Turkey'
- ❑ Spoke to GP / health professional for advice
- ❑ Nicotine replacement therapy
- ❑ Individual counselling/therapy
- ❑ Quitline
- ❑ Other ____________________

Q18 Are you planning to stay off smoking after surgery?

- ❍ Yes
- ❍ No

Q19 What is the likelihood of staying off tobacco cigarettes after hospital?

- ❍ Very likely
- ❍ Likely
- ❍ Undecided
- ❍ Unlikely
- ❍ Very Unlikely

Q20 Do you agree with following statements

	Disagree	Neither Agree nor Disagree	Agree
Smoking causes problems with healing after surgery in smokers	❍	❍	❍
Smoking causes lung problems after surgery in smokers	❍	❍	❍
Smoking causes heart problems after surgery in smokers	❍	❍	❍
Quitting smoking will reduce chances of having problems after surgery	❍	❍	❍

Q21 Electronic cigarettes (e-cigarettes) are electronic devices that deliver nicotine in a vapour and look like cigarettes but contain no tobacco. Have you ever heard of or seen an e-cigarette?

❍ Yes
❍ No

Q22 If you have heard about them or seen them, where did this occur? Tick all that apply?

❑ In-person conversation
❑ Internet
❑ When travelling overseas
❑ TV or radio
❑ Newspapers or magazines
❑ Information shared on social media
❑ Other ____________________

Q23 Have you ever tried an e-cigarette?

❍ Yes
❍ No

Q24 If you have tried an e-cigarette, why?

❑ Curiosity
❑ To try to quit tobacco cigarettes
❑ Easy to use when I can't smoke
❑ Safer than tobacco cigarettes
❑ Cheaper than cigarettes
❑ Other ____________________

Q25 How likely are you to try e-cigarettes in the future?

❍ Strongly Disagree
❍ Disagree
❍ Neither Agree nor Disagree
❍ Agree
❍ Strongly Agree

Q26 I would be willing to try e-cigarettes to help me stay off or cut down regular tobacco cigarettes around the time of surgery

❍ Strongly Disagree
❍ Disagree
❍ Neither Agree nor Disagree
❍ Agree
❍ Strongly Agree

Q27 I think that e-cigarettes could help me stay off or cut down regular tobacco cigarette use around the time of surgery

- ❍ Strongly Agree
- ❍ Agree
- ❍ Neither Agree nor Disagree
- ❍ Disagree
- ❍ Strongly Disagree

Q28 If they were available free of charge, I would try to use them to help stay off or cut down regular tobacco cigarette use around the time of surgery

- ❍ Strongly Agree
- ❍ Agree
- ❍ Neither Agree nor Disagree
- ❍ Disagree
- ❍ Strongly Disagree

Q29 Even if I needed to buy them myself, it would be worth to try e-cigarettes to stay off or cut down regular tobacco cigarettes around the time of surgery

- ❍ Strongly Disagree
- ❍ Disagree
- ❍ Neither Agree nor Disagree
- ❍ Agree
- ❍ Strongly Agree

Q30 Using e-cigarettes instead of smoking regular tobacco cigarettes could help me do better after my surgery

- ❍ Strongly Agree
- ❍ Agree
- ❍ Neither Agree nor Disagree
- ❍ Disagree
- ❍ Strongly Disagree

Q31 E-cigarettes could help me cope with not being able to smoke regular tobacco cigarettes whilst in hospital for my surgery

- ❍ Strongly agree
- ❍ Agree
- ❍ Neither Agree nor Disagree
- ❍ Disagree
- ❍ Strongly Disagree

Q32 It would be better for my health if I could use e-cigarettes around the time of surgery rather than smoking regular tobacco cigarettes
- Strongly agree
- Agree
- Neither Agree nor Disagree
- Disagree
- Strongly Disagree

Q33 Using e-cigarettes could help me improve my health around the time of surgery
- Strongly agree
- Agree
- Neither Agree nor Disagree
- Disagree
- Strongly Disagree

Q34 It would be hard for me to learn how to use e-cigarettes around the time of my surgery
- Strongly Agree
- Agree
- Neither Agree nor Disagree
- Disagree
- Strongly Disagree

Q35 I have too many other things to worry about other than to try e-cigarettes around the time of surgery
- Strongly Agree
- Agree
- Neither Agree nor Disagree
- Disagree
- Strongly Disagree

Q36 E-cigarettes would be too expensive for me to use
- Strongly agree
- Agree
- Neither Agree nor Disagree
- Disagree
- Strongly Disagree

Q37 I am concerned that e-cigarettes are not safe
- Strongly agree
- Agree
- Neither Agree nor Disagree
- Disagree
- Strongly Disagree

Q38 Do you think e-cigarettes or any electronic nicotine delivery systems are allowed to be sold in Australia?

- ❍ Yes
- ❍ No

Q39 Would you try e-cigarettes if they were recommended to you by a health professional, such as your GP or cardiothoracic surgeon, in order to reduce your tobacco smoking now or in the future?

- ❍ Yes
- ❍ No

Appendix B: Interest in, perceived benefits of, and barriers to e-cigarette use (N=62)

	SA	A	NAND	D	SD
**Interest**					
I would be willing to try e-cigarettes to help me stay off or cut down regular tobacco cigarettes around the time of surgery	1 (1%)	21 (34%)	7 (11%)	18 (29%)	15 (24%)
If e-cigarettes were available free of charge, I would try to use them to help stay off or cut down regular tobacco cigarette use around the time of surgery	6 (10%)	18 (29%)	5 (8%)	28 (45%)	5 (8%)
Even if I needed to buy them myself, it would be worth to try e-cigarettes to stay off or cut down regular tobacco cigarettes around the time of surgery	0 (0%)	17 (27%)	7 (11%)	28 (45%)	10 (16%)
I think that e-cigarettes could help me stay off or cut down regular tobacco cigarette use around the time of surgery	4 (6%)	16 (26%)	13 (21%)	21 (34%)	8 (13%)
**Perceived benefits**					
E-cigarettes could help me cope with not being able to smoke regular tobacco cigarettes whilst in hospital for my surgery	3 (5%)	16 (26%)	8 (13%)	29 (47%)	5 (8%)
Using e-cigarettes instead of smoking regular tobacco cigarettes could help me do better after my surgery	4 (6%)	16 (26%)	13 (21%)	21 (34%)	8 (13%)
It would be better for my health if I could use e-cigarettes around the time of surgery rather than smoking regular tobacco cigarettes	3 (5%)	19 (31%)	11 (18%)	22 (35%)	5 (8%)
Using e-cigarettes could help me improve my health around the time of surgery	3 (5%)	14 (23%)	12 (19%)	27 (44%)	6 (10%)
**Perceived barriers**					
It would be hard for me to learn how to use e-cigarettes around the time of my surgery	0 (0%)	10 (16%)	15 (24%)	32 (52%)	5 (8%)
I have too many other things to worry about other than to try e-cigarettes around the time of surgery	3 (5%)	36 (58%)	11 (18%)	10 (16%)	2 (3%)
E-cigarettes would be too expensive for me to use	2 (3%)	14 (23%)	29 (47%)	17 (27%)	0 (0%)
I am concerned that e-cigarettes are not safe	8 (13%)	26 (42%)	13 (21%)	15 (24%)	0 (0%)

Values given as n (%) for the 62 participants. SA: strongly agree; A: agree; NAND: neither agree nor disagree; D: disagree; SD: strongly disagree.

© 2018 Luxton N. A.
